# The Role of Sphingosine-1-Phosphate and Ceramide-1-Phosphate in Inflammation and Cancer

**DOI:** 10.1155/2017/4806541

**Published:** 2017-11-15

**Authors:** Nitai C. Hait, Aparna Maiti

**Affiliations:** Division of Breast Surgery, Departments of Surgical Oncology and Molecular & Cellular Biology, Roswell Park Cancer Institute, Elm & Carlton Streets, Buffalo, NY 14263, USA

## Abstract

Inflammation is part of our body's response to tissue injury and pathogens. It helps to recruit various immune cells to the site of inflammation and activates the production of mediators to mobilize systemic protective processes. However, chronic inflammation can increase the risk of diseases like cancer. Apart from cytokines and chemokines, lipid mediators, particularly sphingosine-1-phosphate (S1P) and ceramide-1-phosphate (C1P), contribute to inflammation and cancer. S1P is an important player in inflammation-associated colon cancer progression. On the other hand, C1P has been recognized to be involved in cancer cell growth, migration, survival, and inflammation. However, whether C1P is involved in inflammation-associated cancer is not yet established. In contrast, few studies have also suggested that S1P and C1P are involved in anti-inflammatory pathways regulated in certain cell types. Ceramide is the substrate for ceramide kinase (CERK) to yield C1P, and sphingosine is phosphorylated to S1P by sphingosine kinases (SphKs). Biological functions of sphingolipid metabolites have been studied extensively. Ceramide is associated with cell growth inhibition and enhancement of apoptosis while S1P and C1P are associated with enhancement of cell growth and survival. Altogether, S1P and C1P are important regulators of ceramide level and cell fate. This review focuses on S1P and C1P involvement in inflammation and cancer with emphasis on recent progress in the field.

## 1. Introduction

Sphingolipids and their derivatives are important structural components of mammalian cell membranes. Sphingolipid metabolites, particularly ceramide, sphingosine-1-phosphate (S1P), and ceramide-1-phosphate (C1P), are lipid mediators that regulate varieties of cellular functions which include cell growth, survival, migration, immune cell trafficking, angiogenesis, inflammation, and cancer [1–3]. It is well established that S1P and C1P are the regulators of sphingolipid rheostat where they reduce proapoptotic ceramide and enhance prosurvival signaling [4, 5]. Inflammation forms the basis of many physiological and pathological processes [6, 7]. Chronic inflammation is associated with asthma, chronic obstructive pulmonary disease (COPD), obesity, type II diabetes, autoimmune disorders, inflammatory bowel disease, and cancer [8, 9]. In response to local tissue damage or infection, neutrophils, macrophages, and other immune cells are recruited to the inflamed tissue from the circulation where they are involved in assisting resolution of inflammation. These processes are marked by the synthesis and secretion of cytokines, chemokines, extracellular matrix proteins, and various lipid mediators including sphingolipid metabolites. Ceramides are the central sphingolipid metabolite known to be part of proapoptotic signaling as well as inflammatory signaling [10–12]. It has been suggested that orosomucoid (ORM) (yeast-) like protein isoform 3 (ORMDL3) gene may be linked with susceptibility to asthma, a chronic airway inflammation and hyperactivity condition [13, 14]. ORMDL3 yeast ortholog is a negative regulator of de novo ceramide biosynthesis [15]. However, we found that high expression of ORMDL3 in lung epithelial cells and macrophages enhances ceramide production, which promoted chronic inflammation, airway hyper responsiveness, and mucus production during house dust mite-induced allergic asthma in a mouse model [16]. Further, nasal administration of the drug FTY720, an immunosuppressant agent, reduced ceramide levels by lowering ORMDL3 expression [16, 17]. In addition, it was found that ORMDL3 also regulates ceramides during IL-1*β*-induced sterile inflammation [17]. Ceramide is enhanced in response to lipopolysaccharide (LPS), saturated fatty acids, or TNF*α*. Ceramide promotes inflammation by varieties of pathways leading to an enhanced effect of obesity [12]. Ceramide stimulates the action of protein phosphatase 2 (PP2), which dephosphorylates AKT [18], decreases survival, and activates Nlrp3 inflammasome to generate active proinflammatory IL-1*β* [19, 20]. Initially, it was experimentally shown that ceramide stimulates Ca^2+^-dependent cytosolic phospholipase A2 (cPLA2) and generates cyclooxygenase 2- (Cox2-) mediated prostaglandins in response to TNF*α* [21]. However, it has been shown that ceramide-1-phosphate (C1P), produced by the ceramide kinase (CERK), activates and translocates cPLA2 more potently than ceramide to generate prostaglandins and inflammatory signaling [22]. Growing evidence and few recent reviews also suggested that sphingosine-1-phosphate (S1P), produced by sphingosine kinases (SphKs), is a progrowth and proinflammatory lipid mediator for cancer progression [10, 23–25]. However, recent data also suggested that both S1P and C1P might have anti-inflammatory roles in certain settings. This review focuses on the current understanding of the role of S1P and C1P in inflammation and cancer.

## 2. Sphingolipid Metabolism

**Figure 1:**
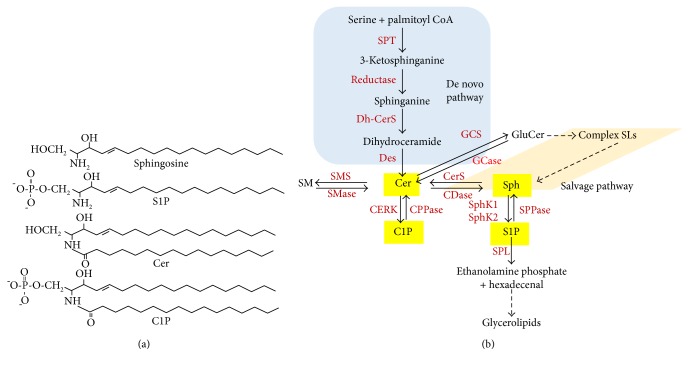
Biosynthesis of ceramide, sphingosine-1-phosphate, and ceramide-1-phosphate (a). Chemical structures of sphingolipids (b). Ceramide is the central sphingolipid molecule of sphingolipid metabolic pathways. Three major pathways are responsible to produce ceramide. Ceramide is produced by de novo pathway in the ER with a series of enzymatic reactions. Ceramide is produce from SM by SMase action. The salvage pathway generates ceramide from sphingosine that generates from the metabolism of complex sphingolipids. Ceramide is now can be converted to C1P by CERK enzyme. Ceramide can be converted to sphingosine by ceramidase. Sphingosine is now phosphorylated by sphingosine kinases to form S1P. S1P can be converted back to sphingosine by SPPase or can be irreversibly broken down by sphingosine phosphate lyase (SPL) to ethanolamine phosphate and 2-trans hexadecenal for phosphatidylethanolamine and glycerolipids, respectively.

**Figure 2:**
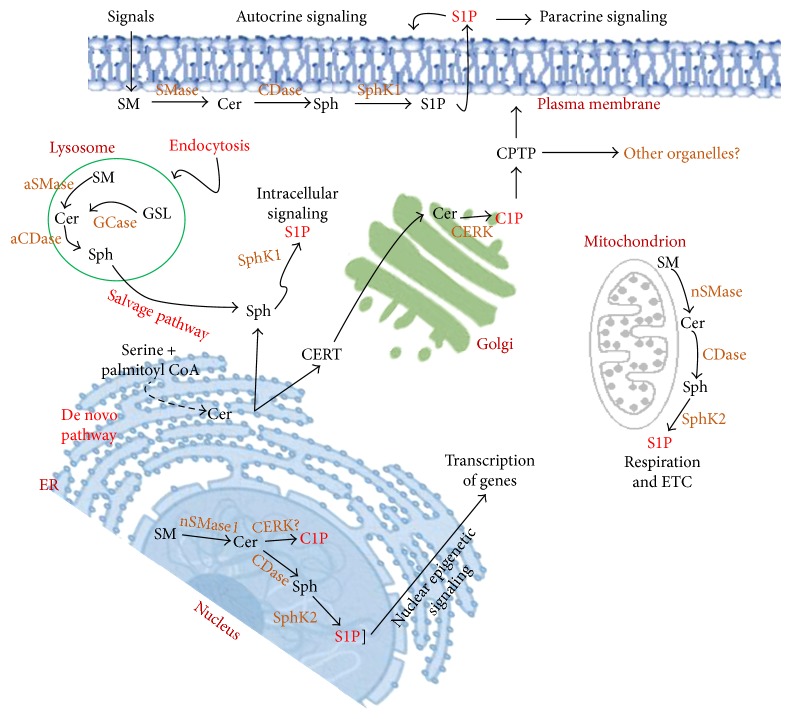
Cellular localization of sphingosine-1-phosphate and ceramide-1-phosphate. ER is the source of ceramide synthesis. Ceramide translocates to the Golgi by ceramide transport protein (CERT) for the synthesis of C1P by Golgi-localized CERK. Ceramide phosphate transfer protein (CPTP) was hypothesized to deliver C1P to the other organelles. Ceramide is produced in the plasma membrane, nucleus, lysosomes, and mitochondria from sphingomyelin (SM). Nuclear CERK can phosphorylate ceramide to form C1P. CERK localization in the mitochondria was not reported. Sphingosine is generated from the ceramide by the ceramidase (CDase) in the plasma membrane, lysosomes, nucleus, and mitochondria. Sphingosine kinase 1 phosphorylates sphingosine to produce sphingosine-1-phosphate (S1P) in the cytosol and plasma membrane for intra- and extracellular signaling. Nuclear and mitochondrial S1P produced from sphingosine by the SphK2 for intracellular signaling.

The de novo synthesis of sphingolipids in the endoplasmic reticulum (ER) starts with the action of serine palmitoyltransferase (SPT) that forms 3-ketosphinganine from serine and palmitoyl coenzyme A (CoA). It has been suggested that SPT activity is negatively regulated by ORMDL proteins [15], which has been identified as a potential risk factor for childhood asthma [14, 16]. 3-Ketosphinganine is converted to sphinganine by a reductase. Ceramide synthase catalyzes the incorporation of an acyl group from fatty acyl-CoA to form dihydroceramide. A desaturase converts dihydroceramide to ceramide by introducing a double bond in positions 4-5 trans (Figure 1). Ceramide is the central sphingolipid of the sphingolipid metabolism. Ceramide is further converted to sphingomyelin by sphingomyelin synthase, to glucosylceramide by glucosylceramide synthase to form complex sphingolipids, to sphingosine by ceramidase, or to C1P by CERK. Sphingosine is further converted to S1P by SphKs. S1P can be converted back to sphingosine by the S1P phosphatase, or it can be irreversibly degraded by S1P lyase to ethanolamine phosphate and hexadecanal (palmitaldehyde). Metabolism of ceramide to complex sphingolipids occurs in the Golgi bodies. Ceramide is delivered to Golgi by ceramide transport protein (CERT) [26]. C1P is formed in the Golgi by CERK [27]. Once the C1P is formed, it is delivered to the plasma membrane for various physiological signaling processes by the C1P transfer protein (CPTP) [28] or CPTP may transfer C1P to other organelles that are not yet known (Figure 2). Recently, it has been shown that phosphatidylserine stimulates C1P intermembrane transfer by CPTP [29]. Endocytic vesicular pathways are involved in plasma membrane complex sphingolipid internalization to the lysosomes where hydrolysis is catalyzed by acid sphingomyelinase (aSMase), acid ceramidase (aCDase), and glycosidase. Finally, sphingosine is formed by a salvage pathway for reutilization into the sphingolipids (Figure 2). SphK1 is mainly a cytosolic lipid kinase. Once activated by various extracellular signaling pathways, it gets phosphorylated by ERK1/2 and translocated to the plasma membrane to form S1P from sphingosine, which functions as an “inside-out” signaling or intracellular signaling molecule for several physiological and pathophysiological processes [1, 30, 31]. On the other hand, SphK2 is localized mainly in the nucleus [32, 33] and partly in the mitochondria [34] to generate S1P from sphingosine at these sites.

Sphingomyelin (SM) and ceramide have also been reported to be present in the nucleus [35–40]. It has been suggested that sphingomyelin synthase (SMS) activity is associated with the nuclear membrane and chromatin of rat liver cells [38, 39]. Nuclear neutral sphingomyelinase-1 (nSMase1) expression has been reported earlier, to metabolize SM to ceramide [40]. Nuclear ceramidase has been shown to metabolize ceramide to form sphingosine [41]. It has been also shown that nuclear localized SphK2 forms S1P from sphingosine [32]. It was thought that CERK at the Golgi synthesizes C1P and CPTP may transfer C1P to the plasma membrane and to the other organelles including nucleus [28]. The CPTP protein is found associated with the plasma membrane, Golgi, and nucleus [28]. It has been shown earlier that CERK is associated with nucleus with its nuclear import signals at the N-terminal and exported to the cytosol with its nuclear export signals at the C-terminal [42]. It was also suggested that the defective nucleocytoplasmic shuttling mechanism of CERK might be responsible for retinal degenerative diseases [42]. Recently, mitochondrial sphingolipid metabolism and its implications to diseases have been described [43]. Ceramide synthase has been detected in the mitochondria, indicating the presence of de novo sphingolipid pathway or salvage pathway to generate ceramide [44]. Mitochondrion-associated nSMase has been identified in the outer membrane of mitochondria [45]. Mitochondrial sphingosine has been shown to form S1P by partially localized SphK2 [34]. An attempt has been taken to measure sphingolipid metabolites in the tissues isolated from human breast cancer patients by using liquid chromatography-electrospray ionization-tandem mass spectrometry methods. Data suggested that levels of sphingolipids in breast cancer tissue are generally higher than normal breast tissue of patients with breast cancer [46].

Taken together, these results suggested that there are tissue and organelle-specific sphingolipid pools that might be potential targets for disease treatments.

## 3. SphK and S1P

S1P is a bioactive lipid mediator for various physiological processes importantly cancer [1, 2, 25, 47]. The major effects of S1P on cancers are summarized in Table 1. S1P is formed intracellularly by two closely related sphingosine kinases: SphK1 and SphK2. SphK1 is a cytosolic protein and may also be localized in the endocytic membrane-trafficking network [48], whereas SphK2 is localized mainly in the nucleus and may be partly localized in the mitochondria of many cells [32, 34]. Both kinases are ubiquitously expressed in all the eukaryotic cells. In most cases, S1P is formed from the cytosolic and is exported from cells by a specific transporter. Extracellular S1P can act on five specific G protein-coupled receptors (S1PR1-5) for its autocrine and paracrine signaling for cancer progression [47, 49]. Cytosolic S1P formed by SphK1 may also act on some recently identified intracellular targets for its involvement in inflammatory signaling pathways before being broken down by S1P lyase. These intracellular targets include TNF receptor-associated factor 2 (TRAF2, an E3 ubiquitin ligase that is a key component of the NF*κ*B pathway [50]; apoptosis inhibitor cIAP2, an E3 ubiquitin ligase that is a key component of the IRF1- (interferon-regulatory factor 1-) mediated immune and sterile inflammation) [51]. Nuclear S1P or its mimetic FTY720-P, generated by SphK2 or enhanced by inhibition of S1P lyase, directly binds to and inhibits class I histone deacetylases (HDACs). This in turn enhances histone acetylation at the promoter of genes that epigenetically regulate gene transcription to promote cancer progression [32, 52–55], regulate lipid metabolisms [56], stimulate memory formation in mice [53], or resolve muscular dystrophy in the dystrophic mouse [52]. The epigenetic effect has recently been identified as a coregulator in a murine model of LPS-induced acute lung injury (ALI) [57]. S1P generated by nuclear SphK2 binds to hTERT allosterically mimic phosphorylation and maintains telomere integrity and stability through limiting proteasome degradation and enhances tumor growth [58]. We have also demonstrated that a fraction of cellular SphK2 is localized to the mitochondrial membrane and produces S1P. Mitochondrial S1P binds to the scaffold protein prohibitin 2, a protein that is important for respiration and the assembly of complex IV. In addition, data from the SphK2^−/−^ mice revealed that S1P is required for ischaemic pre- and post-conditioning cell survival as well as cardioprotection [34, 59]. Mitochondrial S1P also promotes mitochondrial function in dopaminergic neurons of a mouse model of Parkinson's disease [60].

In agreement with previous reports [61] along with our recent study [62], it was suggested that knockdown of SphK2 with siRNA or inhibition of SphK2 activity with the selective pharmacological drugs reduces cancer cell growth, migration, and invasion [61–68], induces apoptosis by accumulating proapoptotic ceramides [63, 64, 69, 70], and promotes proteasomal inhibitor-mediated ER stress resulting in myeloma cell death [71, 72]. In sharp contrast, it has been recently demonstrated that mitochondrial SphK2 is proapoptotic; it produces S1P that is degraded by S1P lyase to hexadecenal, which then binds to the apoptosis regulator BAX, promoting its oligomerization and the release of cytochrome c [73]. However, more studies need to be performed with specific SphK2 inhibitors or mitochondrial targeted SphK2 that would be beneficial to identify clinically relevant functions of SphK2. There are ample evidences suggesting that SphK/S1P signaling pathways are associated with cancer development and metastasis [47]. Overexpression of SphK/S1P signaling is often associated with cancer drug resistance to chemotherapy, radiation therapy, or hormonal therapies in various types of cancers, including breast, prostate, multiple myeloma, and pancreatic cancers [3, 25, 46, 47, 72, 74–77]. Overexpression of SphK1 is associated with poor survival of triple-negative breast cancer patients [78–80]. It has been also shown that estrogen-mediated ER-positive breast cancer cell growth is dependent on SphK1 [62, 81, 82]. Many growth factors, cytokines, and hormones activate SphK1 through phosphorylation at the ser225 residue by active ERK1/2 that facilitates translocation of SphK1 to the plasma membrane. Extracellular S1P activates S1RP3 in ER-positive breast cancer cells to promote tumorigenesis. In ER-negative breast cancer, SphK1 and S1PR4 are associated to promote tumorigenesis. Despite abundant reports strongly suggesting that S1P is associated with cancer progression, few findings obtained with a selective inhibitor of SphK1 or SphK2 however suggested that they are not involved in cell growth of cancer cells [79, 83–86]. SphK1 and SphK2 inhibitors and their effects on cancer are summarized in Table 2. It is important to note that along with SphK1, SphK2 is overexpressed in many human cancers [61, 68, 87–90] and based on its cellular localization it can function as a pro- or antiapoptotic signaling molecule.

FTY720 (fingolimod), an FDA-approved drug for the treatment of multiple sclerosis, has beneficial effects in the CNS that is independent of its effects on immune cell trafficking. We have shown that FTY720 is enriched in the nucleus and phosphorylated by nuclear SphK2 to form FTY720-P. Nuclear FTY720-P binds to and inhibits class I histone deacetylases (HDACs), enhancing specific histone acetylation, and epigenetically enhances gene expression programs associated with memory and learning [53]. Our recent study suggested that nuclear FTY720-P generated from SphK2, acting as a class I HDAC inhibitor, epigenetically reexpressed ER*α* and increased therapeutic sensitivity of ER*α*-negative syngeneic breast tumors to tamoxifen [54], indicating that FTY720 could be a useful anticancer drug. Selective inhibition of SphK2 by the pharmacological inhibitors such as ABC294640 and K145 has shown anticancer effects [70, 91]. Furthermore, a phase I clinical study of ABC294640 in patients with advanced solid tumors has been completed reporting a partial response in a patient with cholangiocarcinoma and stable disease with various solid tumors [92]. Within 12 hours of drug administration, changes of plasma sphingolipids along with decreased level of S1P were observed suggesting that SphK2 is an attractive therapeutic target.

## 4. S1P as a Biomarker in Cancer Progression

There are few recent reports suggesting the role of S1P as a biomarker for cancer progression after measuring the blood levels in human subjects. Plasma S1P levels in ovarian cancer patients were almost twice as high as in healthy controls [93]. Elevated plasma S1P levels were associated with increased risk of developing lung cancer [94]. In contrast, plasma S1P levels of prostate cancer patients were lower than those of age-matched control and this represents an early marker for progression to androgen independence [95]. S1P levels were shown to be also correlated with prostate-specific antigen and lymph node status. The authors suggested that circulating S1P and SphK1 activity in erythrocyte, a major source of blood-borne S1P, are the novel biomarkers for early-stage prostate cancer detection [95]. Recently, major alterations of serum sphingolipid metabolites were investigated in chronic liver disease and were found to be associated with the stage of liver fibrosis in corresponding patients. Serum levels of sphingolipid metabolites showed a significant upregulation in patients with HCC as compared to patients with cirrhosis. It was suggested that particularly C16-ceramide and S1P may serve as novel diagnostic markers for the identification of HCC in patients with liver diseases [96]. In Japanese patients, sphingolipid metabolites, including ceramide and S1P, were measured by LC-ESI-MS/MS comparing normal and breast cancer tissues. Data suggested that the levels of S1P, ceramides, and other sphingolipids in the tumor were significantly higher than the normal breast tissue. It was speculated that the correlation of S1P levels in the breast cancer tissues implies a role of S1P in interaction between cancer and the tumor microenvironment [46]. Another study from the same group also suggested that the levels of S1P in Japanese patients are associated with the clinical parameters in human breast cancer. Levels of S1P in breast cancer tissues were found significantly higher in patients with high white blood cell count in the circulating blood. In contrast, S1P levels were found lower in patients with human epidermal growth factor receptor 2 overexpression and/or amplification. However, there was no difference of S1P levels in the breast cancer tissues based on the expression status of ER or PgR. Another important observation from this study was that patients with lymph node metastasis, one of the major determinants of clinical staging and prognosis, showed significantly higher levels of S1P in tumor tissues than the patients with negative nodes [97]. S1P levels in the breast cancer tissues were correlated with higher expression levels of active SphK1 (S225-pSphK1). However, S1P levels were not associated with tumor size, cancer aggressiveness evaluated pathologically by nuclear grade, cancer cell proliferation quantified by Ki67 staining, or lymphatic invasion [97].

## 5. Role of S1P in Inflammation and Cancer

S1P signaling pathways have been implicated in inflammation and cancer [77, 98]. Many studies have demonstrated that varieties of cytokine and growth factor signaling activate SphK1 and produced S1P that are important for inflammatory processes [1]. In fibroblasts and A549 lung adenocarcinoma cells, S1P induced cycloxygenase2 (COX2) and prostaglandin E2 (PGE2) production [99, 100]. Earlier studies also suggested that basal and activated SphK1 signaling by IL1-*β* and TNF*α* is important for survival and inflammatory signaling in A549 cells [101]. Furthermore, it was shown that S1P-induced COX-2 expression and PGE2 /IL-6 generation were mediated through S1PR1/3/c-Src/PYK2/p42/p44 MAPK- or JNK1/2- and S1PR1/3/c-Src/p38 MAPK-dependent AP-1 activation in human tracheal smooth muscle cells [102]. Additionally, preventing S1P using siRNA against S1P lyase/phosphatase resulted the increased production of COX2 and PGE2 in response to TNF*α* [103], further implicating the key role of S1P in those pathways [103]. More recent studies have suggested that TNF*α*-mediated activation of SphK1 is crucial for TRAF2-mediated K63 polyubiquitylation of RIP1, a key step in NF-*κ*B activation and signaling [50]. However, further studies have demonstrated that SphK1 is not involved in TNF*α*-mediated NF-*κ*B activation; downregulation of SphK1 or SphK1^−/−^ MEFs has rather enhanced CCL5 expression, while downregulation of SphK2 reduced CCL5 expression without affecting NF-*κ*B [104]. However, a recent study also demonstrated that both SphK1 and SphK2 are not required for TNF-mediated NF-*κ*B activation and cytokine expression in mouse macrophages. These cells have increased sphingosine and ceramide levels due to the knockdown of SphKs [105]. The inflammatory role of S1P produced by the two lipid kinases SphK1 and SphK2 in immune cells is not well understood. Some studies using SphK1^−/−^ mice, elegantly reviewed recently [10], suggested that colonic and synovial inflammation is reduced following the knockout mice, whereas other studies with neuroinflammation and lung inflammatory injury by lipopolysaccharide have demonstrated that SphK1^−/−^ mice have increased inflammatory signaling.

The proinflammatory properties of SphK1/S1P are well documented in a TNF*α*-induced inflammatory arthritis mouse model [106–108]. The pro- and anti-inflammatory responses of S1P have been reviewed extensively elsewhere [10, 104, 109]. In immunocompromised mouse xenograft models, it has been shown that selective inhibition of SphK2 diminished NF-*κ*B survival signaling [110], indicating that SphK2/S1P also regulates NF-*κ*B activity and inflammation. A SphK2-deficient MCF-7 breast tumor xenograft mouse model study suggested a role of S1P, generated by SphK2, in early tumor development affecting macrophage polarization [111]. Data suggested that tumor-associated macrophages (TAMs) in the SphK2-deficient tumors displayed a pronounced antitumor phenotype, with an increased expression of proinflammatory markers/mediators such as NO, TNF*α*, IL-12, and MHCII and a low expression of anti-inflammatory IL-10 and CD206 [111]. Potential roles for S1P in the pathophysiology of the liver have been investigated in several studies. S1P has an inhibitory effect on hepatocyte proliferation [112, 113] and a stimulatory effect on hepatic stellate cells [114], which play stimulatory role in hepatic fibrosis [112]. S1P enhances portal vein pressure [115]. Further, it was suggested that increased mRNA expressions of SphK1 and S1P lyase and reduced levels of S1P are associated with progression of hepatocellular carcinoma (HCC) with poorer differentiation and earlier recurrence [116, 117]. The findings suggest that SphK1 and S1P lyases are potential therapeutic targets for HCC treatment. Physiologically, the inflammatory role of S1P and its two kinases is rather complex, cell type specific, and tissue dependent, which requires further detailed studies.

Recent investigation in a kidney fibrosis model of mice revealed that SphK2^−/−^ mice have attenuated kidney fibrosis than wild-type or SphK1^−/−^ littermate mice [118]. SphK2^−/−^ mouse kidneys exhibited greater expression of Interferon (IFN) and IFN-gamma-responsive genes (Cxcl9 and Cxcl10) than those of WT or SphK1^−/−^ mice. This could be due to the compensatory mechanism of SphK1 or due to the anti-inflammatory effect of S1P. Another interesting study demonstrated that SphK2 might be a key component for the facilitation of nociceptive circuits in the CNS leading to central sensitization and pain memory formation [119].

It has long been known that S1P is involved at multiple stages of the asthmatic responses. Inhalation of SphK1 selective inhibitor or FTY720 attenuates airway inflammation in an asthmatic mouse model [120, 121]. In mast cells, S1P produced by the SphKs contributes to inflammatory and allergic responses [122]. Exogenous S1P-stimulated production and secretion of cytokines, like TNF*α* and IL-6, markedly enhanced the secretion of chemokines, like CCL2/MCP-1, which are important modulators of inflammation [123]. Further studies suggested that S1P/S1PR2 axis regulates early airway T-cell infiltration in murine mast cell-dependent acute allergic responses [124]. In sterile inflammation, it is well established that IRF1 (interferon-regulatory factor 1) is essential for IL-1-induced expression of the chemokines CXCL10 and CCL5, which recruit mononuclear cells into sites of sterile inflammation. Intracellular S1P synthesized by SphK1 was required to activate the apoptosis inhibitor cIAP2 for Lys63- (K63-) linked polyubiquitination of newly synthesized IRF1 and chemokine synthesis [51]. This study further strengthens the fact that S1P is important for IL1-*β*-mediated sterile inflammatory signals. Our recent work in Duchenne muscular dystrophy (DMD) model suggested that delivery of 2-acetyl-5-tetrahydroxybutyl imidazole (THI), a S1P lyase inhibitor, suppresses dystrophic muscle degeneration. The THI effect further correlated with significantly increased nuclear S1P, decreased HDAC activity, and increased acetylation of specific histone residues in mdx mice. Furthermore, gene expression analysis revealed a significant THI-dependent decrease in inflammatory genes and an increase in metabolic genes associated with the mitochondrial function [52].

It has been suggested that S1P is a procancer signaling molecule for various types of cancer [47, 61, 125]. Using a SphK1^−/−^ mouse model, it has been demonstrated that S1P generated by SphK1 promotes pancreatic cancer progression [126]. SphK1/S1P is also involved in chronic intestinal inflammation-associated cancer [127, 128]. Mice lacking intestinal S1P lyase exhibited greater disease activity of colitis-associated cancer (CAC); these include colon shortening, increase of cytokine levels, S1P accumulation, tumor formation, STAT3 activation, STAT3-activated microRNAs (miRNAs), and suppression of miR-targeted antioncogene products [107, 108]. These studies clearly suggested that S1P is a pro-inflammatory molecule enhancing inflammation-associated colon cancer. We have shown that SphK1 is linked with chronic intestinal inflammation to colitis-associated cancer in a mouse model. SphK2^−/−^ mice have high expression of SphK1 in the colon tissues and in the circulation. SphK2^−/−^ mice showed an exacerbated effect of CAC. Further, SphK1 was linked with NF-*κ*B-regulated cytokine IL-6, persistent activation of STAT3, and consequent upregulation of the S1P receptor, S1PR1. We have shown that FTY720 decreased SphK1 and S1PR1 expression and eliminated the NF-ĸB/IL-6/STAT3 amplification cascade and development of CAC [128]. Together, these data suggested that targeting S1P signaling might represent a novel strategy in treating inflammation-associated colon cancer.

## 6. CERK and C1P

CERK directly phosphorylates ceramide to form C1P. Its activity is regulated in response to IL-1*β* and calcium ionophore A23187 leading to stimulation of arachidonic acid release and subsequent generation of proinflammatory eicosanoids in A549 lung adenocarcinoma cells [129, 130]. This further suggested C1P as a novel regulator of cell activation [131]. CERK activity was initially detected in brain tissue [132] and found to have been ubiquitously expressed in all the mammalian cells. CERK is a 60 kDa lipid protein that contains N-terminal myristoylation and pleckstrin homology (pH) domains, which are required for association with cell membranes [130]. Further research suggested that CERK is localized to the trans-Golgi networks with its pH domain and utilizes ceramide as a substrate which is transported from the ER to the Golgi by the ceramide transport protein (CERT) [28]. Once C1P is formed in the Golgi, it can be transferred to the plasma membrane by a specific C1P transfer protein (CPTP) [28], probably for its unidentified autocrine and paracrine signaling. It has been implicated that determinants for localization of CERK are not solely dependent on its N-terminal pH domain region. It has been reported that mutation in the pH domain also destabilizes the enzyme. In addition, leucine 10 in the pH domain of the CERK seems to play an important role in regulating its enzymatic activity [133]. CERK activity is regulated by tyrosine kinase-mediated pathway, implying active phosphorylation and dephosphorylation mechanisms to regulate CERK functions [134]. Another interesting observation suggested that agonists of nuclear receptor peroxisome proliferator-activated receptors (PPARS), particularly PPARbeta and PPARdelta, protect neural cells against ceramide-induced cell death via induction and activation of CERK [135], indicating CERK involvement in neurodegenerative diseases. All-trans retinoic acid (ATRA) is an active metabolite of vitamin A. Retinoids, through their cognate nuclear receptors, exert potent effects on cell growth, differentiation, and apoptosis and have a significant promise for cancer therapy and chemoprevention [136]. It has been suggested that ATRA downregulated CERK mRNA level during ATRA-induced differentiation of human neuroblastoma cells. ATRA inhibited transcriptional activity of CERK via regulation of a COUP-TF1 transcription factor, indicating that CERK/C1P might be an important lipid signaling molecule for cancer cell survival [137]. The hormonally active metabolite of vitamin D, 1,25-dihydroxyvitamin D3, is an important regulator of cell growth and differentiation. 1,25-Dihydroxyvitamin D3 has been shown to potently inhibit CERK activity, thus reducing cancer cell growth, again indicating that CERK is a survival kinase for cancer cells [138]. Atopic dermatitis (AD) is a chronic, allergic, and inflammatory skin disease associated with eczema and dermatitis symptoms. It has been suggested that eriodictyol, a bitter-masking flavanone extracted from Yerba Santa (*Eriodictyon californicum*), potently inhibits CERK expression and improves atopic dermatitis, a chronic, allergic, and inflammatory skin disease in a mouse model [139]. Past few studies have demonstrated that CERK activation and intracellular C1P are involved in noncancer and cancer cell growth and survival. [140–144]. Macrophage-colony stimulating factor (M-CSF) activates CERK and produces intracellular C1P that is important for its mitogenic effect on macrophages through activation of the PI3-kinase/PKB, JNK, and ERK1/2 pathways [144]. Exogenous C1P has been shown to stimulate macrophage motility by a pertussis-toxin-sensitive GPCR [142], indicating that an extracellular cell surface receptor of C1P might be involved in cell migration. Recent studies have shown that exogenous C1P-mediated cell migration was shown dependent on Gi protein-coupled receptor, indicating unidentified cell surface C1P receptor involvement in this process [141].

CERK has also been found to be overexpressed in breast cancer and associated with poor prognosis [145, 146]. CERK promotes tumor cell survival and mammary tumor recurrence [147, 148]. Originally, CERK/C1P has been shown to enhance lung cancer cell growth and survival [140]. It has been demonstrated that CERK/C1P is involved in pancreatic cancer cell migration and invasion, and survival is dependent on phosphatidylinositol 3-kinase (PI3K) and ROCK1 pathways [141]. C1P has been explained to promote migration of hematopoietic cells and released as an antiapoptotic molecule when cells are damaged. It is also reported that C1P regulates migration of multipotent stromal cells and endothelial progenitor cells to the damaged organs that may promote their vascularization [149], suggesting the role/function of C1P similar to S1P in regenerative medicine [150]. C1P also has been shown important for priming of mesenchymal stromal/stem cells (MSCs) by enhancing their migratory, self-renewal properties that have implications in pulmonary artery hypertension patients [151]. Like S1P, C1P is involved in trafficking of normal stem cells and cancer cells may have implications in tumor microenvironment and prevention of cancer metastasis [152]. Both S1P and C1P are strongly enhanced the *in vitro* motility and adhesion of human rhabdomyosarcoma (RMS) cells [153]. Gamma-irradiation or chemotherapy treatment increased levels of S1P and C1P in several organs suggesting their association in prometastatic microenvironment [153]. CERK/C1P is also an important inducer for proliferation of renal mesangial cells [154], suggesting that CERK inhibition may have therapeutic potential.

## 7. Role of C1P in Inflammation and Cancer

Originally, it was demonstrated that ceramide kinase (CERK) produces its product C1P inside the cells and C1P is the mediator of arachidonic acid (AA) released in cells in response to interleukin-1*β* and calcium ionophore [129]. Later, it was found that C1P is a direct activator of group IV cytosolic phospholipase A2 (cPLA2) [22]. The role of sphingolipids in cPLA2-mediated AA synthesis and their involvement in inflammatory disorders have been studied extensively [155, 156]. Particularly, it has been implicated that CERK and C1P are required to activate, as well as translocate cPLA2 from cytosolic compartment to intracellular membranes such as Golgi bodies to form AA, which is the substrate for COX2 to form prostanoids in the A549 human lung carcinoma cell line [22]. Prostanoids are a subclass of eicosanoids consisting of the prostaglandins, the thromboxanes, and the prostacyclins, involved in inflammatory processes with roles in the pathogenesis of cancer and inflammatory disorders. The COX-2 pathway of prostanoid synthesis has already been established as an important therapeutic target for the treatment of inflammatory disorders [157, 158]. Ceramide activates cPLA2 that activates AA release and is involved in COX2-mediated inflammation. Further, it was demonstrated that ceramide is a more potent activator of cPLA2 for AA release and Cox-2-mediated PGE2 formation compared to C1P [99]. It arrears that C1P with acyl chain length of 6 carbons or more in length is potent to activate cPLA2 in *in vitro* enzyme assay condition [159]. In addition to the direct interaction of C1P to the cPLA2, it has been shown that the activity of PKC isoforms *α* and *δ* is involved in C1P-mediated AA release in murine fibroblasts [160]. Ubiquitously expressed lipid transfer protein (CPTP) was shown to transfer C1P between membranes [28]. Crystal structure analysis demonstrated the specific binding of C1P with CPTP [28]. It has been implicated that CPTP is a cytosolic protein but is associated with Golgi bodies and plasma membrane. It transfers C1P from trans-Golgi network to plasma membrane and may be to other organelles [28]. Interestingly, depleting CPTP with siRNA elevates steady-state level of C1P in the Golgi network and stimulates cPLA2 alpha-mediated AA release to activate proinflammatory eicosanoid production [28]. These observations suggested that targeting C1P level at the Golgi complex potentially targets cPLA2-mediated eicosanoid synthesis and related proinflammatory pathological processes [28]. Interestingly, S1P has been shown to mediate the effect of cytokines on COX2 activation and PGE2 production which implicated that both S1P and C1P are acting coordinately for COX2-mediated eicosanoid synthesis and inflammatory responses [99]. C1P increases specifically the transport of P-glycoprotein, an ATP-driven efflux pump which regulates the permeability of the blood-brain barrier (BBB) via COX2/PGE2 signaling [161], which offers clinical benefits for drug delivery to the CNS to modulate neuroprotection [161].

In postoperative ileus inflammation which is characterized by intestinal dysmotility, both C1P and S1P levels are elevated in smooth muscle cells in a rat model [162]. Another interesting study explained that CERK and its product C1P are involved in wound healing process, implicating that mechanical scratch wound stimulated C1P, that enhanced AA-mediated eicosanoid synthesis for inflammatory responses in the fibroblast isolated from CERK^+/+^ mice to higher level than in fibroblasts derived from CERK^−/−^ mice [163]. Proper migration of fibroblasts is the important process of wound healing; as expected, it was observed that CERK and its product C1P were absolutely required for migration of fibroblast for wound healing [163]. CERK has been speculated to be highly expressed in the CNS (including the spinal cord) [164]. Pharmacological inhibition of CERK ameliorated the chronic inflammatory phase of pain induced by a s.c. injection of formalin on the dorsal side of the hind paw in rats [164, 165], indicating that CERK might have a contribution to inflammatory pain. CERK has been shown to regulate TNF-stimulated NADPH oxidase activity and eicosanoid biosynthesis in neuroblastoma cells, suggesting its critical role in CNS inflammation [166].

In addition to these inflammatory processes, C1P has been implicated in calcium-dependent degranulation and inflammatory processes in mast cells [167–169]. However, it has been demonstrated using bone marrow-derived mast cells (BMMC) isolated from CERK^−/−^ mice that CERK is not essential for mast cell activation but it might act as a calcium sensor [170].

Although it has been proposed that CERK and C1P/cPLA2 activation could be a therapeutic target for PGE2 involved inflammatory diseases [171]; however, understanding related to C1P mediated cPLA2 involvement in cytokine synthesis is still lacking. Murine arthritis inflammation model has demonstrated that CERK^−/−^ mice are not protected compared to its wild-type counterparts given the fact that cPLA2 is an important part of this model [172]. It might be possible that C1P/cPLA2-mediated inflammation is cell type specific [173] as it was originally been demonstrated in A549 lung epithelial cancer cells.

Inflammatory mechanisms are linked with obesity [174] and associated with the production of proinflammatory cytokines such as IL-6 and TNF*α* [175, 176]. It was observed that deletion of CERK suppressed high-fat diet obesity-mediated inflammatory cytokines IL-6 and TNF*α* and showed normal insulin signaling in an animal model [177]. CERK also has been shown to regulate biogenesis of lipid droplets [178]. It is well documented in the literature that macrophage infiltration into adipose tissue is a hallmark in obesity-evoked inflammation [143]. By using a high-fat diet obesity mice model, it has also been demonstrated that CERK^−/−^ mice have reduced macrophage infiltration and MCP-1 signaling in the adipose tissue, resulting in attenuation of inflammatory responses [177]. Surprisingly, CER^−/−^ animals still have significant amount of C1P indicating that there might be alternative pathways to account for the C1P in these animals [179, 180]. Although such alternative pathways of C1P synthesis could include cleavage of SM by phospholipase D type SMase (SMase D) activity or transfer of fatty acyl chain to S1P for the synthesis of C1P [181], these pathways remain to be discovered.

Recently, C1P in the pathogenesis of cigarette smoke-triggered pulmonary inflammation and emphysema in humans and mice has been identified. C1P potently inhibits cigarette smoke-associated airway inflammation. Specifically, C1P inhibited both acute and chronic inflammation and attenuated the development of emphysema potently in a mouse model of chronic obstructive pulmonary disease (COPD) [182]. Evidence suggested that C1P may have anti-inflammatory properties depending on cell types and tissues. Anti-inflammatory action of C1P in this COPD model was associated with inhibition of the activity and expression of N-SMase, NF-*κ*B, and the proinflammatory cytokines TNF*α*, IL-1*β*, IL-6, keratinocyte chemoattractant (KC), and macrophage inflammatory protein-2 (MIP-2) in mouse lungs and human airway epithelial cells and neutrophils [182]. Earlier studies on macrophages have also demonstrated that exogenous C1P acts as an anti-inflammatory regulator of TNF*α* production and NF-*κ*B expression in response to lipopolysaccharide (LPS) [183, 184]. More recent studies also support the fact that exogenous C1P signaling acts as anti-inflammatory pathways in LPS-induced acute lung injury mouse model. It has been shown that exogenous C1P in both *in vivo* and ex vivo models attenuates LPS-induced lung injury by preventing NF-*κ*B activation and IL-8 production in human neutrophils [185]. However, natural sphingolipid C1P stimulates macrophage function and migration, whereas synthetic C1P mimic (PCERA-1) suppresses production of TNF*α* but enhances anti-inflammatory cytokines such as IL-10 in response to LPS [186]. This study conveys that exogenous natural sphingolipid C1P and synthetic C1P mimic may act on macrophages via distinct different cell surface receptors [186]; however, further studies are required to clarify. Exogenous C1P causes upregulation of metalloproteinases (MMP)-2 and −9 in J774A.1 macrophages via PI3K and ER1/2 pathways [187]. It is established that acid sphingomyelinase (A-SMase) and downstream ceramides are important players for chronic inflammation of the airways associated with chronic obstructive pulmonary disease (COPD) [188]. It is possible that inhibition of A-SMase and subsequent depletion of ceramide levels by CERK to form C1P may be beneficial to cure lung inflammatory diseases. Recently, pro- and anti-inflammatory properties of exogenous C1P are nicely reviewed by many investigators [148, 179, 181]. Previously, Mitra et al. [140] reported that exogenous C1P at low concentrations enhanced survival and proliferation of NIH3T3 fibroblasts and A549 lung cancer cells while at high concentrations reduced survival and induced apoptosis that is correlated with degradation of C1P to proapoptotic ceramide [4, 140]. Moreover, CERK is involved in cell cycle progression induced by epidermal growth factor (EGF) in lung cancer cells via activation of ERK1/2 [140]. Following this study many research supported the fact that CERK/C1P is an important component of survival signaling for cancer progression [3, 141, 152, 181, 189–191]. Commercially available ceramide kinase inhibitor NVP-231 inhibits breast and lung cancer cell proliferation by inducing M phase arrest and subsequent cell death [146]. CERK signaling has been shown important for human pancreatic cancer migration and proliferation suggesting that it is an important pharmacological target for controlling pancreatic cancer [141]. Multiple studies have suggested that PI3K/AKT and Ras/Raf/MEK/ERK pathways are involved in CERK/C1P-mediated cell survival [142, 148, 189]; however, detailed molecular mechanism of CERK-mediated cell migration, proliferation, and invasion is not well understood. Gene expression profiles from more than 2200 patients revealed that elevated CERK expression is associated with an increased risk of recurrence in women with breast cancer [147]. This study was further validated in a mouse model and supported that CERK/C1P is important for breast cancer recurrence. Studies from the same group along with others supported that CERK expression is associated with high grade aggressive basal and HER2^+^ breast cancer subtypes [147]. It appears that like S1P, CERK/C1P is also involved in proinflammatory signaling and cancer progression. Although C1P in certain scenarios acts as an anti-inflammatory molecule, but in most part, it is speculated that CERK may be a target for a new anti-inflammatory drug and probably for inflammation-associated cancer.

## 8. Conclusions

Although the physiologic roles S1P and C1P are not fully understood, most evidences suggested that S1P and C1P are important molecules in inflammation and cancer. The discovery of intracellular targets of S1P along with its extracellular signaling will provide broad spectrum of research opportunities to identify the role of S1P as an anti- or proinflammatory signaling molecule. The epigenetic role of nuclear sphingolipids will allow the understanding of the transcriptional regulation of the synthesis of inflammatory cytokines or chemokines. Further research is required to demonstrate the organelle-specific role of sphingolipids, which might enlighten additional knowledge to understand their role in inflammation and cancer. The discovery of new cell surface receptors for C1P or new organelle-specific intracellular targets of C1P will identify their precise role in inflammation and cancer.

## Figures and Tables

**Table 1:** Major effects mediated by S1P and C1P in cancer.

Lipids	Mechanism	Functions	References
S1P	(i) Intracellularly, generated S1P secreted out of the cancer cells by ABCC1 transporter. Extracellular S1P is a ligand for G protein coupled receptors S1PR1-5 (ii) Intracellular S1P binds and modulates E3 ubiquitin ligases activity. Mitochondrial S1P binds to prohibitin 2 (PHB2) and regulates complex IV assembly and respiration. Nuclear S1P binds and inhibits histone deacetylase 1 and 2 (HDAC 1 and 2) and epigenetically regulates histone acetylation and transcription of genes associated with cancer progression	(i) Tumor progression (ii) Metastasis (iii) Cancer cell survival (iv) Cell migration (v) Angiogenesis (vi) Inflammation (vii) Chemokine signaling (viii) Immune cell trafficking (ix) Epigenetic regulation	This manuscript and [145]

C1P	(i) Extracellular C1P is a ligand for unidentified G protein-coupled receptor (ii) Intracellular C1P binds and activates cPLA2*α* (iii) Intracellular C1P binds CPTP and vesicular trafficking	(i) Tumor progression (ii) Metastasis (iii) Cancer cell survival (iv) Migration and invasion (v) Inhibition of apoptosis (vi) Inflammation (vii) Eicosanoid synthesis (viii) Macrophage functions	This manuscript and [32, 50, 51, 54, 62, 81, 128]

**Table 2:** SphK1 and SphK2 inhibitors and their effects in cancer.

SphK inhibitors	Selectivity	Cancer type	References
SKI-1	SphK1	Breast cancer, glioblastoma, leukemia, colon cancer	[3, 28, 99, 141, 146, 147, 152, 154, 186]
K-145	SphK2	Leukemia, breast cancer	[81, 128, 192]
PF-543	SphK1	Breast, colon, and colorectal cancer, leukemia	[62, 91]
ABC294640	SphK2	Liver, breast (ER^+^, ER^−^), pancreas, bladder, prostate, colorectal, colitis-driven colon, and ovarian cancer, phase I advanced solid tumors, multiple myeloma, cholangiocarcinoma, lung cancer	[62, 83, 193–195]
SKI-II and ABC294735	SphK1 and SphK2	Kidney and pancreatic adenocarcinoma	[66, 70, 92, 196–203]
DMS	SphK1 and SphK2	Breast, lung, and colon cancer, hepatocellular carcinoma, gastric cancer	[204, 205]
SG-12 and SG14	SphK2	Cervical cancer	[62, 206–209]
Safingol	SphK1 and SphK2	Phase I with cisplatin in advanced solid tumors	[65, 210]

## Abbreviations

S1P: Sphingosine-1-phosphate
C1P: Ceramide-1-phosphate
SphK: Sphingosine kinase
CERK: Ceramide kinase
CTP: Ceramide transfer protein
CPTP: Ceramide-1-phosphate transfer protein
Cer: Ceramide
Sph: Sphingosine
ER: Endoplasmic reticulum
PM: Plasma membrane
SPT: Serine palmitoyltransferase
ORMDL3: ORM1-like protein 3
Dh-CerS: Dihydroceramide synthase
Des: Desaturase
SMS: Sphingomyelin synthase
SM: Sphingomyelin
SMase: Sphingomyelinase
nSMase: Neutral sphingomyelinase
A-SMase: Acid sphingomyelinase
CPPase: Ceramide phosphate phosphatase
GCase: Glucosylceramidase
CDase: Ceramidase
CerS: Ceramide synthase
SPPase: Sphingosine phosphate phosphatase
SPL: Sphingosine phosphate lyase
GPCR: G protein couple receptor
PGE2: Prostaglandin E2
Cox2: Cyclooxygenase 2
cPLA2*α:*: Cytosolic phospholipase A2
TNF*α:*: Tumor necrosis factor *α*
IL: Interleukin.
